# Association Between the rs13306703 and rs8192288 Variants of the *SOD3* Gene and Breast Cancer and an In Silico Analysis of the Variants’ Impact

**DOI:** 10.3390/diseases12110276

**Published:** 2024-11-02

**Authors:** Martha Patricia Gallegos-Arreola, Asbiel Felipe Garibaldi-Ríos, María Teresa Magaña-Torres, Luis E. Figuera, Belinda Claudia Gómez-Meda, Guillermo Moisés Zúñiga-González, Ana María Puebla-Pérez, Irving Alejandro Carrillo-Dávila, Mónica Alejandra Rosales-Reynoso, Ingrid Patricia Dávalos-Rodríguez, Jorge I. Delgado-Saucedo, Marco Uriel López-Monroy

**Affiliations:** 1 División de Genética, Centro de Investigación Biomédica de Occidente, Centro Médico Nacional de Occidente, Instituto Mexicano del Seguro Social, Sierra Mojada 800, Col. Independencia, Guadalajara 44340, Jalisco, Mexico; asbiel.garibaldi4757@alumnos.udg.mx (A.F.G.-R.); maganamt@gmail.com (M.T.M.-T.); luisfiguera@yahoo.com (L.E.F.); irving.carrillo4754@alumnos.udg.mx (I.A.C.-D.); ingrid.davalos@academicos.udg.mx (I.P.D.-R.); 2 Doctorado en Genética Humana, Centro Universitario de Ciencias de la Salud, Universidad de Guadalajara, Guadalajara 44340, Jalisco, Mexico; 3 Instituto de Genética Humana “Dr. Enrique Corona Rivera”, Departamento de Biología Molecular y Genómica, Centro Universitario de Ciencias de la Salud, Universidad de Guadalajara, Guadalajara 44340, Jalisco, Mexico; belinda.gomez@academicos.udg.mx; 4 División de Medicina Molecular, Centro de Investigación Biomédica de Occidente, Centro Médico Nacional de Occidente, Instituto Mexicano del Seguro Social, Sierra Mojada 800, Col. Independencia, Guadalajara 44340, Jalisco, Mexico; mutagenesis95@hotmail.com (G.M.Z.-G.); mareynoso@hotmail.com (M.A.R.-R.); 5 Laboratorio de Inmunofarmacología, Centro Universitario de Ciencias Exactas e Ingenierias, Universidad de Guadalajara, Guadalajara 44430, Jalisco, Mexico; ana.puebla@academicos.udg.mx (A.M.P.-P.); jorge.delgado@academicos.udg.mx (J.I.D.-S.); 6 Maestría en Ciencias en Química, Centro Universitario de Ciencias Exactas e Ingenierías, Departamento de Química, Universidad de Guadalajara, Guadalajara 44430, Jalisco, Mexico; marco.lopez1369@alumnos.udg.mx

**Keywords:** breast cancer, *SOD3* gene, genetic variants, cancer genetics, in silico analysis

## Abstract

**Background/Objectives:** This study investigated the association between the rs13306703 and rs8192288 variants of the superoxide dismutase 3 (*SOD3*) gene and breast cancer (BC) in the Mexican population, conducting both genetic and in silico analyses. **Methods:** 357 healthy women and 386 BC patients were studied using TaqMan assays, qPCR, and RFLP-PCR. **Results:** The *TT* genotype and a recessive pattern of these variants were risk factors for BC (*p* < 0.05). Specifically, the *TT* genotype of rs13306703 was associated with metastatic lymph nodes, tumor progression (III–IV), luminal A, nonresponse to chemotherapy, and ki-67 ≥ 20% with diabetes mellitus (DM). Meanwhile, the *GT* genotype of rs8192288 was associated with menopause, luminal A, tumor progression (III–IV), ki-67 ≥ 20%, and a positive estrogen receptor with nonresponse to chemotherapy. Additionally, the *TT* genotype combined with DM was identified as a BC risk factor (*p* < 0.05). The *TT* haplotype was also found to be a risk factor for BC. In silico analysis suggested that these variants might influence *SOD3* regulation by affecting transcription factors and active enhancer sites. **Conclusions:** The rs13306703 and rs8192288 variants of the *SOD3* gene were associated with an increased risk of BC and may alter *SOD3* regulation through effects on transcription factors, active enhancers, and transcription start sites, with modified motifs in breast epithelium cells.

## 1. Introduction

Breast cancer (BC) is the most common type of cancer in women worldwide and is one of the leading causes of death among women. The latest GLOBOCAN [1] report indicates that BC is the most common type of cancer among Mexican women, with an incidence of 39.9 cases and a mortality rate of 10.3 per 100,000. Although it is a heterogeneous and multifactorial disease, molecular and genetic factors play an important role in its development [2,3]. Oxidative stress, characterized by an imbalance between the production of reactive oxygen species (ROS) and the body’s antioxidant system to neutralize them, has been described as playing an important role in developing BC and other types of neoplasms [4,5,6,7]. ROS induces DNA damage, stimulating the carcinogenic process by promoting genomic instability, modifying gene expression patterns, and encouraging mutations. Thus, cell proliferation and the invasion and metastasis of cancer cells are favored [8,9,10]. In BC, this process is relevant due to the susceptibility of breast cells to mutations in DNA repair genes, such as *BRCA1* and *BRCA2*. In BC, ROS also affects hormonal pathways, facilitating tumor growth [11]. Antioxidant enzymes, such as those belonging to the superoxide dismutase (SOD) family, including SOD1, SOD2, and SOD3, play a crucial role in regulating oxidative stress [12]. SOD3, also known as extracellular superoxide dismutase, is the only enzyme in the SOD family that performs its function outside the cell. This enzyme catalyzes the conversion of superoxide anion (O_2_−) into oxygen (O_2_) and hydrogen peroxide (H_2_O_2_) [12,13,14]. Encoded by the *SOD3* gene and located on chromosome 4, SOD3 contains two exons and one intron [15]; it is composed of 240 residues and has specific regions that allow it to bind to the extracellular matrix. This feature is crucial for its protective function [16,17]. Recently, decreased expression of *SOD3* has been observed in several types of cancer, including lung cancer [18], as well as in highly invasive tumor cells compared to those with low invasiveness [19]. Furthermore, it has been demonstrated that the expression of SOD3 enhances therapeutic response in tumor-associated endothelial cells [20]. Additionally, its expression has also been implicated as an inhibitor of cancer cell migration in the thyroid tumor stroma [21]; however, studies on the expression of this gene in BC have not yet been published.

Variants of the *SOD3* gene are associated with an increased risk of cervical cancer [22], prostate cancer [23], gliomas [24], BC [25], stroke [17], and emphysema in obstructive pulmonary disease [26]. Some variants of the *SOD3* gene likely contribute to a decrease in its expression, which could be related to cancer. Studying these variants is crucial for understanding how genetic variations in the *SOD3* gene are associated with BC risk since these variants may influence the gene’s expression, function, or activity [17,18,19,20,21,22,23,24,25,26]. In this study, two variants of the *SOD3* gene were analyzed: rs13306703 and rs8192288.

According to Ensembl data [15] (Ensembl.org, accessed 4 June 2024), these variants are located in intronic regions. The SNV (single nucleotide variant) variant rs13306703 shows a *C*>*T* change, while rs8192288 is multiallelic, with the *G* allele being the most frequent and the *A* and *T* alleles being the alternative alleles. Variants in introns, although not located in coding regions, can significantly impact gene expression and their association with cancer. These variants can affect RNA splicing sites, altering the correct elimination of introns and the binding of exons, which can result in abnormal or nonfunctional proteins. In addition, they can influence the modification of intronic regulatory sequences that control transcription and gene expression [16,27,28,29,30]. In this study, we investigated the frequency and association of the variants rs13306703 and rs8192288 with BC in Mexican women and performed an in silico analysis to predict their possible biological or regulatory impact. It is important to note that these variants have not yet been analyzed in the context of BC or in the Mexican population.

## 2. Materials and Methods

### 2.1. Study Population

DNA genomic samples from 386 Mexican patients clinically and pathologically confirmed with BC and 357 DNA samples from healthy donors from the Mexican population were included in this study. The patient group comprised women aged 18 years or older with any stage of BC, regardless of treatment status or therapeutic stage. The healthy donor group consisted of healthy female donors, also aged 18 years or older, from the general Mexican population. The guidelines provided in the Declaration of Helsinki were followed to ensure the welfare and rights of the study participants. All patients were informed about the objectives and procedures of the study, and their written informed consent was obtained before sample collection. The study protocol was evaluated and approved by the local ethics committee under registration number R-2021-1305-006 at the Centro de Investigación Biomédica de Occidente, Instituto Mexicano del Seguro Social (CLIES #1305), ensuring compliance with all relevant ethical and legal regulations.

### 2.2. Variant Analysis

**Figure 1:**
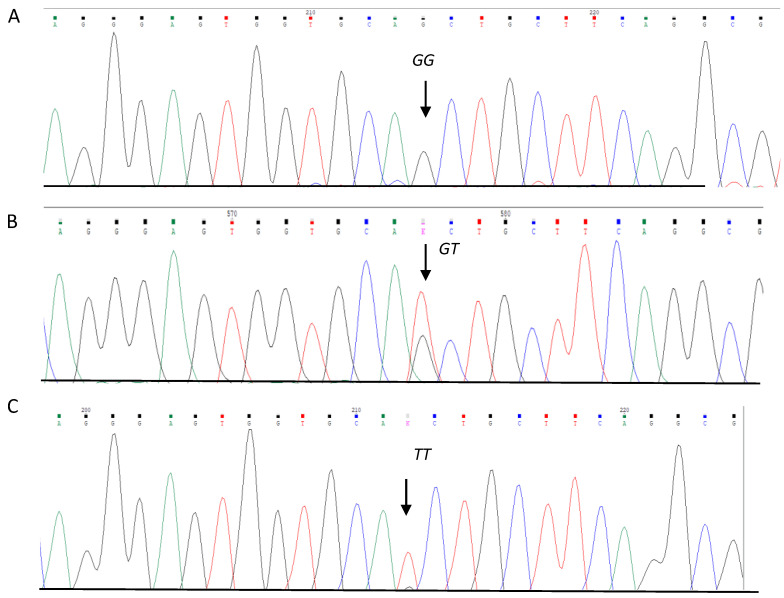
Identification of rs8192288 variant in the *SOD3* gene sequence. (**A**) *GG* wild-type homozygous, (**B**) *GT* heterozygous, and (**C**) *TT* variant homozygous.

The rs13306703 variant was genotyped using TaqMan qPCR assays designed and validated by Thermo Fisher Scientific, ensuring accurate and efficient detection of this variant. The sequence design of the probes is as follows: [VIC/FAM]. ATGGTGGGGGGAGGTTGGGGGGGCGGTGG[C/T]GAGAAAAAGGCAGATTTCCTCTAGA (C__30938535_10). To genotype the variants, the samples were analyzed in a final volume of 10 μL and read using a CFX96 real-time PCR system (Bio-Rad Laboratories, Berkeley, CA, USA) together with the fluorescent probes described previously. The rs8192288 variant was analyzed by PCR using the following primers: F 5′-GGAAACACTCCTAGTTCTC-3′ and R 5′-CATGGAAATGGGCACCTTGC-3′ (selected from https://www.ensembl.org/Homo_sapiens/Gene/Sequence?db=core;g=ENSG00000109610;r=4:24789912-24800842) (accessed on 3 October 2022). The PCR reaction contained 0.25 mM dNTPs (Invitrogen, Carlsbad, CA, USA), 5 pmol of primer, 3.0 mM MgCl2, 1 μL of DMSO, 2.5 U of Taq polymerase (Invitrogen, Carlsbad, CA, USA), and 50 ng of genomic DNA in a total volume of 15 μL. The annealing temperature was 59 °C. The PCR product was digested with a PvuII restriction enzyme. The 392 base pair (bp) PCR products were separated on 6% polyacrylamide gels (29:1), followed by silver staining. The *G* allele (wild type) had a PvuII cleavage site and was digested into 154 and 238 bp fragments, while the *T* allele (variant) had no recognition for the restriction site and was identified with 392 bp fragment. To ensure the accuracy of the results, 10% of the reactions were repeated for analysis as an internal control by Sanger sequencing by capillary electrophoresis using a SeqStudio Sequencer and BigDyerR Terminator v3.1 Cycle Sequencing kit (Thermo Fisher Scientific Inc., Waltham, MA, USA). The *GG*, *GT*, and *TT* genotypes of the rs8192288 variant were identified (Figure 1).

### 2.3. In Silico Analysis

#### 2.3.1. Prediction of the Regulatory Role of the Analyzed Variants

To predict the possible biological or regulatory impact of the variants, we used the RegulomeDB platform [31] (https://regulomedb.org/ accessed on 10 June 2024) and HaploReg [32] (https://pubs.broadinstitute.org/mammals/haploreg/haploreg.php accessed on 11 June 2024).

These are bioinformatics tools that provide functional interpretations of variants located in noncoding regions. They integrate data from assays such as TF ChIP-seq and QTL, offering a comprehensive range of genomic regulatory information, including promoter activity, enhancers, and transcription factor binding.

#### 2.3.2. Analysis of SOD3 Expression in BC

We analyzed *SOD3* expression profiles in tissue samples from BC patients using data obtained from the Xena platform [33] (https://xenabrowser.net accessed on 11 June 2024) and OncoDB [34] (https://oncodb.org/ accessed on 11 June 2024) platform. For this analysis, we compared the mean expression in BC samples (*n* = 1135) to the mean expression in tissue from healthy patients (*n* = 114). The expression data were provided by the platforms and pre-normalized to transcripts per million (TPM) by RNA-Seq. The Xena and OncoDB platforms provide access to gene expression and clinicopathological data from projects such as the Cancer Genome Atlas (TCGA) and offer interactive tools for visualization.

### 2.4. Statistical Analysis

Allele and genotypic frequencies were determined by direct counting. The Hardy–Weinberg equilibrium was calculated to ensure that the allele frequencies of the reference group were in equilibrium. Subsequently, SPSS Statistics Base 24 statistical software (IBM Corp., Armonk, NY, USA) was used to compare the genotypes amongst study subjects, calculate binary odds ratios (logistic regression), and assess the associations between BC and the clinicopathological features of the disease. In addition, the SHEsis platform was utilized to calculate linkage disequilibrium and observed haplotype frequencies [35]. A *p*-value of <0.05 was considered significant for all statistical tests performed.

## 3. Results

### 3.1. General Characteristics of the Study Groups

The average age of the patients with BC was 50.32 ± 12.93 years, while the average age of the control group was 50.23 ± 12.64. There were no significant differences between the two groups (*p* > 0.05) (Table 1). The clinical data for BC patients revealed the following: menopause status (212/386, 55%), unilateral tumor localization (363/386, 94%), ductal type (355/386, 92%), stage II (154/386, 40%), luminal A subtype (124/386, 32%), luminal B subtype (8/386, 23%), Her-2 positive (51/386, 13%), triple-negative (123/386, 32%), ki-67 ≥ 20% (263/386, 68%), chemotherapy responders (164/386, 43%), and gastric toxicity to chemotherapy (209/386, 54%) (Table 1).

### 3.2. Genotype Analysis of rs13306703 and rs8192288 Variants of SOD3

The *TT* genotype of the rs13306703 variant was associated with an increased risk of BC, with a frequency and recessive model showing an odds ratio (OR) 2.17 (95% confidence interval [CI] 1.46–3.23, *p* = 0.0001). Similarly, the *T* allele of the rs8192288 variant (OR 1.40, 95% CI 1.09–1.80, *p* = 0.009), along with the *TT* genotype frequency and the recessive model (OR 2.95, 95% CI 1.51–5.77, *p* = 0.001), were identified as risk factors for BC (Table 2). Additionally, the additive model for the rs13306703 variant was not statistically significant, but the rs8192288 variant showed an OR of 1.40 with *p* < 0.05.

In the BC group, genotype *TT* of the rs13306703 variant was associated with several risk factors: metastatic lymph nodes (OR 1.7, 95% CI 1.03–2.9, *p* = 0.038), advanced stage (III–IV) (OR 2.6, 95% CI 1.05–6.6, *p* = 0.039), luminal A type (OR 1.7, 95% CI 1.03–2.9, *p* = 0.039), and nonresponse to chemotherapy (OR 1.7, 95% CI 1.07–2.7, *p* = 0.043) with metastatic lymph nodes. Additionally, ki-67 ≥ 20% combined with diabetes mellitus (DM) was also identified as a risk factor (OR 3.0, 95% CI 1.2–4.7, *p* = 0.018) (Table 3).

The *GT* genotype of the rs8192288 variant was associated with the following risk factors: menopausal hormonal status; luminal A type with menopausal hormonal status (OR 2.4, 95% CI 1.04–4.6, *p* = 0.040); advanced stage (III–IV) with ki-67 ≥ 20% (OR 3.0, 95% CI 1.4–4.6, *p* = 0.003); and positive estrogen receptor with nonresponse to chemotherapy (OR 2.0, 95% CI 1.02–4.2, *p* = 0.043) (Table 2). The *TT* genotype was associated with DM (OR 2.2, 95% CI 1.03–4.7, *p* = 0.040) as a risk factor. The dominant model *GTTT* was associated with the presence of DM in luminal A type (OR 2.9, 95% CI 1.37–4.3, *p* = 0.027) and nonresponse to chemotherapy (OR 2.3, 95% CI 1.1–3.7, *p* = 0.021) as risk factors (Table 3).

Comparisons of the studied groups showed statistically significant differences in the frequency of the *TT* haplotype (OR 4.0, 95% CI 1.92–8.59, *p* = 0.0001), identifying it as a risk factor for BC (Table 4). The linkage disequilibrium analysis of rs13306703 and rs8192288 revealed a D’ of 0.28 and r^2^ = 0.01.

### 3.3. In Silico Analysis

#### 3.3.1. *SOD3* Expression in BC

**Figure 2:**
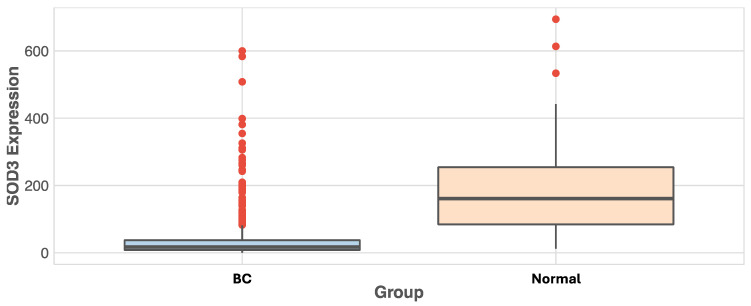
Differential expression of *SOD3* in BC and healthy controls.

The in silico expression analysis revealed a decrease in *SOD3* expression in BC, with a mean expression of 33.8 in BC patients and a mean expression of 182.1 in healthy controls (logFC −3.24, *p* = 5.6 × 10^−23^) (Figure 2).

#### 3.3.2. Regulatory Role of the Analyzed Variants

**Figure 3:**
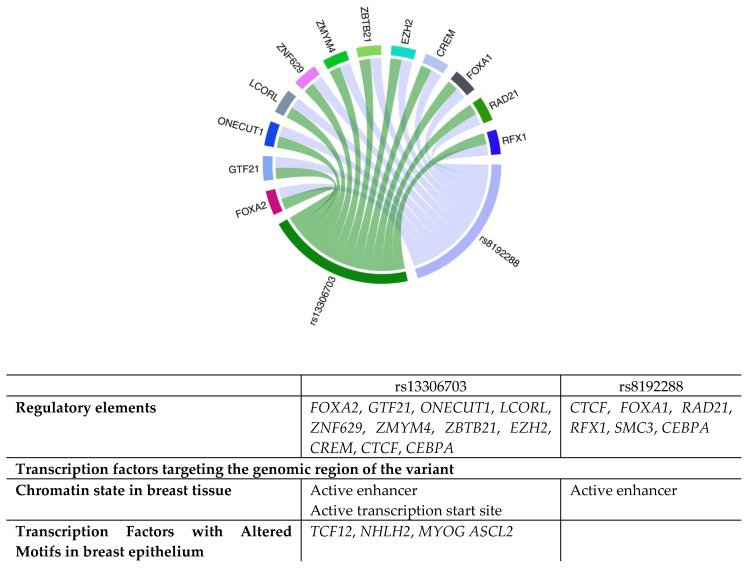
Regulatory elements affected by the rs13306703 and rs8192288 variants of the *SOD3* gene.

According to the data obtained from the in silico tools, the rs13306703 variant is classified as rank 4 in RegulomeDB, suggesting that it could significantly influence gene regulation. This ranking is based on the presence of transcription factors and a chromatin accessibility peak in the region where the variant is located. In addition, the variant has a regulatory score of 0.70497, indicating a high probability that it could alter *SOD3* activity as it is in a crucial region for the regulation of this gene (Figure 3).

On the other hand, the rs8192288 variant has a rank 2b classification, suggesting that the variant could notably affect gene regulation. This ranking is based on multiple signals of importance, including the presence of transcription factor (TF) binding, the coincidence with a motif recognized by these factors, evidence of a protein footprint in the DNA, and localization to a chromatin accessibility peak. Chromatin accessibility peaks are regions where transcription factors and regulatory proteins interact with DNA to regulate gene expression. Thus, a variant in this region may alter the binding of these regulatory factors.

These features suggest that the variant could significantly influence gene activity by modifying the interaction of transcription factors with DNA, which is crucial for precisely regulating gene expression. Furthermore, the variant has a regulatory score of 0.79882—a value of 0 indicates a minimal probability of being a regulatory variant, and a value of 1 represents the maximum probability of being a regulatory variant. This score further strengthens the evidence that the rs8192288 variant could have a significant impact on the regulation of gene activity. Figure 3 details the regulatory characteristics of the analyzed variants.

#### 3.3.3. Regulatory Pathway of the rs13306703 and rs8192288 Variants of the *SOD3* Gene

**Figure 4:**
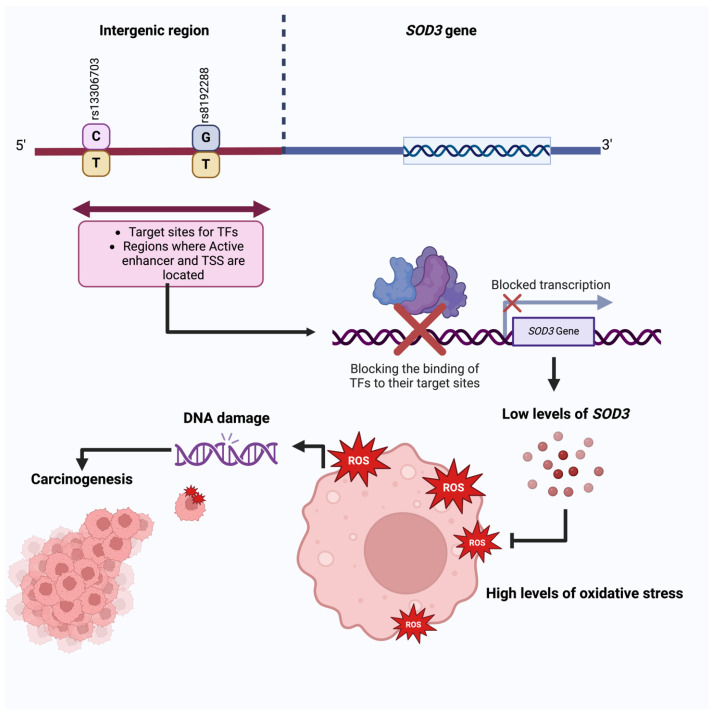
Regulatory role of the variants. The analyzed variants, located within critical regulatory elements, may lead to decreased expression of *SOD3*. Reduced *SOD3* levels can increase oxidative stress within cells, resulting in oxidative damage to DNA and other biomolecules. This pro-oxidative environment can induce genetic mutations and epigenetic alterations that promote malignant transformation. These molecularly intricate events establish a tumor microenvironment conducive to cancer initiation, progression, and treatment resistance. Created with Biorender.com.

The preceding in silico analysis of the regulatory roles of these variants showed that they are situated at the binding sites of diverse transcription factors, including active enhancers and TSS. This indicates their potential to modulate regulatory functions by potentially obstructing these elements and consequently reducing *SOD3* transcription levels. A schematic depiction of the regulatory pathway influenced by the analyzed variants is provided in Figure 4.

## 4. Discussion

BC is one of the main causes of death from tumors in women in Mexico and around the world [1,36]. In our study, we found that the rs13306703 and rs8192288 variants of the *SOD3* gene are associated with an increased risk of developing BC in the Mexican population as well as with certain clinicopathological features of the disease.

The average age of the participants in this study was approximately 50 years, which is consistent with previous reports [2,36] and highlights other related risk factors characteristic of BC, including menopause, ductal type histology, stage II BC, luminal A, triple negative, and nonresponse to chemotherapy. Despite various BC cancer prevention campaigns in Mexico, the number of women under 50 years of age with this type of cancer continues to increase [2,36,37]. Therefore, it is important to conduct more studies on the Mexican population to understand their genomics and the biological mechanisms of BC.

Concerning the regulation process of oxidative stress in the tumor microenvironment, different cellular mechanisms related to the participation of *SOD3* have been proposed [13,14,16,18,25]. The function of *SOD3* in the tumor microenvironment is uncertain. The negative regulation of *SOD3* in a tumor is associated with its progression through the pro-oncogenic NF-κB and HIF-1α signaling pathways, as has been observed. It participates in apoptosis induced by DNA damage, and in breast tissue, it is involved in the activation of vascular endothelial growth factor (VEGF) through the kinase pathway. However, in some benign tumors, *SOD3* is positively correlated, and in mouse embryonic fibroblast cells, high levels of the enzyme promote cell growth and transformation [38]. *SOD3* is also inhibited in some solid tumors through promoter hypermethylation and histone modification mechanisms called epigenetic silencing; mutations in the promoter or heparin-binding domain of the *SOD3* gene are driven by oncomiR-21. The location of the *SOD3* gene on chromosome 4 (4p15.1–4p15.3) is considered a critical point for the loss of heterozygosity (LOH) in cancer [38]. It has antitumor effects by altering the structure, composition, and dynamics of the extracellular matrix mediated by ROS and by inhibiting metalloproteinase activity, such as heparinase, which promotes angiogenesis, proliferation, and invasion of cancer by inducing the degradation of the sulfated glycosaminoglycan heparan sulfate [39]. Therefore, *SOD3* could act as an intrinsic and extrinsic tumor suppressor of cancer cells [38].

In this study, we observed that the frequency of the *TT* genotype of both the rs13306703 and rs8192288 variants showed statistically significant differences between the BC patients and the controls (*p* < 0.05) and were associated with an increased risk of developing BC. These variants have not been studied in other populations with BC or other cancers, so our findings cannot be compared. It should be noted that this is the first study conducted in the Mexican population in which variants of the *SOD3* gene are analyzed in relation to BC.

Similar to our study, previous research [26,40,41,42] has also demonstrated the association of the rs13306703 and rs8192288 variants of *SOD3* with various non-neoplastic conditions, including cerebral infarction, essential hypertension, and chronic obstructive pulmonary disease. These findings suggest that these variants may play a broader role in disease susceptibility by potentially influencing pathways related to oxidative stress and inflammation, which are relevant not only for cancer development but also for these other conditions.

On the other hand, association studies on different cancers focusing on other variants of the *SOD3* gene have shown contradictory results. For example, variants that have demonstrated an association with susceptibility to risk include the rs2536512 variant (G172A) in cervical cancer in women from Maharashtra [22] and the rs699473 variant in brain tumors [43]. However, variants rs1799895 (–896*C*>*G*) and rs2536512 were not shown to be associated with colon or gastric cancer, respectively [44,45].

One of the mechanisms proposed for the association between *SOD3* and cancer is through the accumulation of circulating ROS, which generates an imbalance of *SOD3* in the cell, cellular signaling, and transcription factors and gives rise to tumorigenesis [5,16,17,18,19,20,21,22,23,24,25,38,46]. Low levels of *SOD3* expression have been observed in lung and prostate tumor tissue [18,47], while high expression of *SOD3* in lung tumor tissue was associated with a low probability of survival due to the infiltration of the proteins PDCD1 (programmed cell death 1) and CTLA4 (lymphocyte-associated protein 4 T cytotoxic), which play a role in the immune system [47].

Clinical variables in the BC patient group were associated with risk susceptibility with the *TT* genotype of the rs13306703 variant. While carriers of the *GT*, *TT,* or *GTTT* genotypes of the rs8192288 variant were associated with metastatic lymph nodes, their combination was linked to progression (stage III–IV), luminal A, and non-response to chemotherapy.

Different molecular mechanisms of *SOD3* have been proposed for the development of carcinogenesis in breast tissue [5,7,10,46,48]. Regarding the association of *SOD3* with clinical features in BC patients, various mechanisms have been proposed to explain the role of *SOD3* in the extracellular space, including signal transduction, tumor suppression, stimulation of signaling networks, immunomodulation, regulation of angiogenesis, anti-inflammatory properties, and mechanisms that induce cell growth and affect various biological processes. These suggest that the loss of intracellular expression of *SOD3* promotes a microenvironment conducive to tumor growth by giving a selective advantage to tumor cells [49,50,51].

It has been shown that in menopausal women with advanced stages of cancer, luminal A, estrogen receptors, and DM, who are non-responders to chemotherapy, there is a large generation of free radicals. One study revealed that low levels of antioxidant enzymes such as *SOD3* are associated with the proliferation of tumors [5,29,46,48]. Therefore, many intrinsic factors in the tumor microenvironment can affect SOD expression levels, such as inflammation, oxidative stress, and altered cellular metabolism [5,49,51]. A previous study revealed the suppression of *SOD3* expression levels in mammary tumor tissue taken from rats treated with estrogen, suggesting that this molecule may play an important role in the prevention of breast cancer and that the *SOD3* gene could have significant potential in developing therapeutic strategies for the prevention of estrogen-induced neoplasia [52]. *SOD3* also participates in the basement membrane of the tumor vasculature, and through the WNT signaling pathway, it causes extravasation of effector T cells in the tumor microenvironment and regulates the density of tumor-infiltrating lymphocytes in primary human colorectal cancers (CRC), affecting relapse rates and patient survival [38,44].

It has been shown that the specific re-expression of *SOD3* in tumor endothelial cells (VEC and HIF-2α) increases the delivery of doxorubicin and enhances the chemotherapeutic effect in tumors [20]. The administration of polynitroxylabumin (PNA, also known as VACNO) mimics SOD3 in triple-negative BC and has been shown to increase survival and reduce lung metastasis [53]. The VEGF-C-Sod3 axis has been shown to play an important role in BC, with *SOD3* being a critical mediator of VEGF-C-induced metastasis [54]. *SOD3* also participates in the epigenetic mechanism of methylation, where a predominantly downregulated expression pattern of *SOD3* and various genetic and epigenetic deregulations suggest that the loss of this antioxidant promotes a microenvironment advantageous for tumor development in BC [55]. *SOD3* is significantly decreased in BC, and its mRNA expression is inversely correlated with relapse-free survival in BC patients. Conversely, lower levels of methylation have been observed as a characteristic of the luminal B subtype; the −78 CpG site has been identified as the most significantly methylated [55]. Furthermore, copy number variation analysis from the TCGA database revealed that the more aggressive triple-negative and HER2 + subtypes had higher levels of *SOD3* gene deletion [55].

Several biomarkers of oxidative stress are known to be altered at the onset of diabetes, and it has been suggested that a low SOD3 concentration provides evidence for reduced extracellular antioxidant defense against superoxide in the early development of DM. It has also been observed that increased oxidative stress is involved in the pathogenesis of experimental diabetic neuropathy [56]. Ki-67 is considered a marker of cellular proliferation in BC and other types of cancer; one study revealed that low levels of antioxidant enzymes, such as SOD, in tumors are associated with tumor proliferation [26,46,48]. In addition, in the current study, the haplotype association of the rs13306703 and rs8192288 of the variants of the *SOD3* gene was determined in BC patients and the control group. The haplotypes showed no linkage disequilibrium with each other. We observed that the *T/T* haplotype was associated with susceptibility to BC; however, it should be noted that the confidence intervals were high due to the small sample size.

To our knowledge, this is the first study to report this association in Mexican BC patients. However, we emphasize that the progression of cancer is associated with adverse clinical outcomes and may modify the expression of different molecular factors, including stress oxidative mechanisms. These changes could alter the regulation of cellular processes and depend on the interaction of several genes involved in multiple metabolic pathways and epigenetic events [46,48]. On the other hand, it has been noted that circulating levels of SOD3 increase when BC patients respond to treatment with a reduction in tumor size during neoadjuvant chemotherapy. Therefore, it is inferred that in patients who do not respond to chemotherapy, the expression levels of SOD in the tumor microenvironment are low, which may accelerate tumor growth [57].

### In Silico Analysis

Our in silico expression analysis revealed a decrease in *SOD3* expression in BC, which is consistent with what has been described in the literature [13,14,18,25,28,46,50]. Using an open-access database, 15 regulatory motifs were identified in transcription factors of the rs13306703 and rs8192288 variants (FOXA2, FOXA1, GTF21, ONECUT1, LCORL, ZNF629, ZMYM4, ZBTB21, EZH2, CREM, CTCF, CEBPA, RAD21, RFX1, and SMC3) at binding sites of diverse transcription factors, including active enhancers and TSS. This indicates their potential to modulate regulatory functions by potentially obstructing these elements, consequently reducing *SOD3* transcription levels. Additionally, altered motifs in transcription factors within the breast epithelium (TCF12, NHLH2, and MYOG ASCL2) were also identified.

In this regard, FOXA1 (HNF3α, hepatocyte nuclear factor 3α) and FOXA2 (Forkhead Box A2), as part of the forkhead box family, are critical in regulating hormone responses and metabolism. FOXA1 is essential for estrogen receptor activity and mammary gland development, while FOXA2 is involved in glucose metabolism and lipid regulation [58,59].

The *GTF2I* gene (general transcription factor III) encodes two proteins, TFII-I and BAP-135, which play roles in gene regulation, growth, and immune responses. It is significant for DNA binding and immune system activation. The BAP-135 protein, active in B cells, triggers chemical reactions to produce antibodies against viruses. The gene promotes the formation of functional ARID3A DNA-binding complexes and the activation of immunoglobulin heavy chain transcription upon B cell activation [60]. The *ONECUT1* (one cut homeobox 1) gene is a DNA consensus sequence-binding transcription factor activator of RNA polymerase II-specific DNA-binding transcription, influencing glucose metabolism and cancer cell cycle regulation [61]. The *LCORL* (ligand-dependent nuclear receptor corepressor-like) gene encodes a transcription factor that binds to DNA elements and plays a role in spermatogenesis, skeletal structure, and stature [62]. ZNF629 (zinc finger protein 629) is a transcription factor that activates DNA-binding transcription specific to RNA polymerase II and RNA polymerase II cis-regulatory sequences [63]. The *ZMYM4* (zinc finger MYM-type containing 4) gene allows DNA binding and is involved in cytoskeleton organization and cell regulation [64]. ZBTB21 (zinc finger and BTB domain containing 21) is involved in the negative regulation of transcription by RNA polymerase II and allows binding to POZ and methyl-CpG domains [65].

The *EZH2* gene (enhancer of zeste 2 polycomb repressive complex 2 subunit) enables binding to specific DNA sequences and chromatin. It belongs to the polycomb group family of genes, is involved in maintaining gene repression, and has been linked to poor prognosis in triple-negative breast cancer due to its role in chromatin modification and signaling pathways [66]. In triple-negative BC, overexpression of the *EZH2* gene correlates with splenic peritoneal progression and poor prognosis [67]. CREM (CAMP responsive element modulator) is involved in the cAMP-dependent activation of the PKA pathway, DREAM repression, and dynorphin expression. It functions as a DNA-binding transcription factor with core promoter sequence-specific DNA sites and encodes the bZIP transcription factor that binds to promoter regions [68]. *CTCF* (CCCTC-binding factor) is a member of the BORIS + CTCF gene family and encodes a transcriptional regulatory protein with 11 highly conserved zinc finger (ZF) domains. This protein binds to different histone acetyltransferases and functions as either a transcriptional activator or a component of a histone deacetylase–containing complex. CTCF, as a transcriptional repressor, has been associated with various cancers. It plays an important role in chromatin remodeling during mitosis and participates in methylation processes [69].

The *RAD21* (RAD21 cohesin complex component) gene is essential for DNA repair and chromatid cohesion and crucial for maintaining genomic stability [70].

The *RFX1* (regulatory factor X1) gene encodes a transcription factor involved in immune responses and cancer, including the regulation of MHC class II genes [71]. The *SMC3* (structural maintenance of chromosome 3) gene participates in chromosome cohesion and signaling pathways, critical for cell cycle progression [72]. The *TCF12* (transcription factor 12) gene is involved in NTRK signaling and DNA binding, impacting gene transcription related to cancer progression [73].

NHLH2 (nescient helix-loop-helix 2) is involved in protein dimerization and the binding of DNA-specific transcription factors to RNA polymerase II. It plays a role in the regulation of transcription by RNA polymerase II [74]. *MYOG* (myogenin) participates as a key factor in muscle development and differentiation. *MYOG* also plays a role in regulating muscle-specific genes. [75]. ASCL2 (achaete-scute family BHLH transcription factor 2) is involved in embryonic and induced pluripotent stem cells, lineage-specific markers, and human early embryo development. It includes DNA-binding transcription factor activity and sequence-specific DNA binding of the cis-regulatory region of RNA polymerase II. ASCL2 is a member of the basic helix–loop–helix (BHLH) family of transcription factors, activating transcription by binding to the E-box (5′-CANNTG-3′). Efficient DNA binding requires dimerization with other BHLH proteins [76].

In silico analyses, integrated with the complete sequencing of the human genome, have led to large-scale cancer studies that have enhanced our understanding of cancer mechanisms and the tumor environment. These studies have helped verify existing hypotheses and generate new ones, highlighting the usefulness and ongoing improvement of tools such as TCGA and PCAWG [77,78].

We recognize that this study has limitations that may impact the interpretation and generalizability of the results. Firstly, the absence of comparative studies in other populations constrains the ability to extend our findings broadly. Although in silico analysis suggests reduced *SOD3* gene expression in BC, it would be beneficial to perform direct gene expression analyses in our population and investigate whether the studied variants affect this expression and contribute to breast cancer risk. Despite these limitations, our findings offer valuable insights into the genetic factors associated with BC in the Mexican population. The identification of key variants and their potential role in BC susceptibility highlights the need for further research to validate and expand upon these results. Such studies could enhance our understanding of the genetic underpinnings of BC and inform more personalized approaches to prevention and treatment.

## 5. Conclusions

Our results showed that the *TT* genotype and recessive model of both the rs13306703 and rs8192288 variants were associated with an increased risk of BC when compared with the control group. Furthermore, significant differences were observed in BC patients stratified by the *TT* genotype of the rs13306703 variant, including the presence of metastatic lymph nodes combined with progressive tumor stages (III–IV), luminal A type, nonresponse to chemotherapy, and ki-67 ≥ 20% with DM. The rs8192288 variant was also identified as a risk factor for BC patients carrying the *GT*, TT, and *GTTT* genotypes, particularly when combined with menopause, luminal A type, advanced tumor progression (stages III–IV), ki-67 ≥ 20%, ER-positive, nonresponsiveness to chemotherapy, and DM. Additionally, the presence of the *TT* haplotype was associated with increased susceptibility to BC.

Moreover, the study demonstrated decreased levels of *SOD3* expression in BC. The importance of this work lies in its identification of 15 regulatory elements and transcription factors that target the genomic regions of the variants, including active enhancers, active transcription starts sites, and transcription factors with altered motifs in the breast epithelium. These elements interact with the analyzed *SOD3* gene variants in silico, highlighting different signaling pathways. Further studies are needed to confirm the findings.

## Figures and Tables

**Table 1:** Socio-demographic and clinical features of the study groups.

		BC Patients (n = 386)		Controls (n = 357)		*p* Value
**Age (years, average ± SD)**		50.32 ± 12.93		50.23 ± 12.64		0.925 *
		n	%	n	%	
**≤49 years**		170	44.0	(157)	44.0	1.0 **
**≥50 years**		216	56.0	(200)	56.0	
**Hormonal status**	premenopause	174	45.0			
	menopause	212	55.0			
**Tumor localization**	unilateral	363	94.0			
	bilateral	23	6.0			
**Histology (adenocarcinoma)**	ductal	355	92.0			
	Lobular	27	7.0			
	Mixed	4	1.0			
**Stage**	In situ	11	3.0			
	I	39	10.0			
	II	154	40.0			
	III	120	31.0			
	IV	62	16.0			
**Molecular Type**	Luminar A	124	32.0			
	Luminar B	88	23.0			
	Her-2	51	13.0			
	Triple-negative	123	32.0			
**Ki67**	≥20%	263	68.0			
	<20%	123	32.0			
**Chemotherapy status**	Response	222	58.0			
	No response	164	42.0 ***			
**Toxicity**	Gastric	209	54.0			
	Hematologic	56	15.0			
	Both	121	31.0			

* Student’s *t*-test; ** Chi-square test, *** Includes non-response, partial response, and non-response due to recurrence.

**Table 2:** Genotype and allelic distribution of the rs13306703 and rs8192288 variants of the *SOD3* gene in study groups.

Variants			BC		Controls *		OR	95% CI	*p*-Value
rs13306703	Model	Genotype	(n = 386)	%	(n = 255)	%			
		*CC*	(158)	41	(92)	36	1.0		1.0
		*CT*	(117)	30	(122)	48	0.46	0.33–0.63	0.0001
		*TT*	(111)	29	(41)	16	2.10	(1.41–3.14)	0.0001
	Dominant	*CC*	(158)	41	(92)	36			
		*TT* + *CT*	(228)	59	(163)	64	0.81	(0.59–1.12)	0.217
	Recessive	*TT*	(111)	38	(41)	23	2.10	(1.41–3.14)	0.0002
		*CC* + *CT*	(275)	62	(214)	77			
		Alleles	(2n = 772)		(2n = 510)				
	Additive						2.85		0.3994
	Alleles	*C*	(433)	0.560	(306)	0.600	0.86	(0.67–1.06)	0.165
		*T*	(339)	0.440	(204)	0.400	1.17	(0.93–1.47)	0.165
rs8192288		Genotype	(n = 386)	%	(n = 357)	%			
		*GG*	(238)	62	(239)	67	1.0		1.0
		*GT*	(112)	29	(106)	30	0.96	(0.70–1.32)	0.903
		*TT*	(36)	9	(12)	3	2.95	(1.51–5.77)	0.001
	Dominant	*GG*	(238)	62	(239)	67			
		*TT* + *GT*	(148)	38	(118)	33	1.25	(0.93–1.70)	0.133
	Recessive	*TT*	(36)	9	(12)	3	2.95	(1.51–5.77)	0.001
		*GG* + *GT*	(350)	91	(345)	97			
	Additive						1.40		0.0392
		Alleles	(2n = 772)		(2n = 714)				
		*G*	(588)	0.762	(584)	0.182	0.71	(0.55–0.91)	0.009
		*T*	(184)	0.238	(130)	0.182	1.40	(1.09–1.80)	0.009

OR (odds ratio), CI (confidence intervals, and *p*-value (significant < 0.05). * Hardy–Weinberg equilibrium for the control group: variants rs13306703 (chi-square test = 0.002; *p* = 0.96), and rs8192288 (chi-square test = 0.003, *p* = 0.95).

**Table 3:** Variants rs13306703 and rs8192288 of the *SOD3* gene and their association with the clinic-pathological features in the BC group.

Variant	Genotype	Variable	OR	95% CI	*p*-Value
rs13306703	*TT*	Metastatic lymph nodes	1.7	1.03–2.9	0.038
		III-IV stage, and metastatic lymph nodes	2.6	1.05–6.6	0.039
		Luminal A, and metastatic lymph nodes	1.7	1.03–2.9	0.039
		Non-response to chemotherapy, and metastatic lymph nodes	1.7	1.07–2.7	0.043
		Ki-67 (≥20%), and the presence of DM	3.0	1.2–4.7	0.018
rs8192288	*GT*	Menopause status	2.4	1.04–4.6	0.040
		Luminal A, and menopause status	2.4	1.04–4.6	0.040
		III-IV stage and Ki-67 (≥20%)	3.0	1.4–4.6	0.003
		Estrogen receptor-positive, and non-response to chemotherapy	2.0	1.02–4.2	0.043
	*TT*	DM	2.2	1.03–4.7	0.040
	*GTTT*	Luminal A, and DM	2.9	1.37–4.3	0.027
		DM, and non-response to chemotherapy	2.3	1.1–3.7	0.021

OR (odds ratio), CI (confidence intervals, *p*-value (significant <0.05).

**Table 4:** Haplotype frequency of the rs13306703 and rs8192288 variants of the *SOD3* gene in studied groups.

Haplotype		BC ^(2n = 740)^		Controls ^(2n = 357)^			
rs13306703	rs8192288	(n)	%	(n)	%	OR 95%[CI]	*p*-Value
*C*	*G*	(310)	42	(161)	45	1	1
*C*	*T*	(105)	14	(57)	16	0.87 [0.61~1.23]	0.504
*T*	*G*	(262)	35	(132)	37	0.93 [0.72~1.21]	0.683
*T*	*T*	(63)	9	(8)	2	4.0 [1.92~8.59]	0.0001

## Data Availability

Data and materials are available in the article.
